# Pediatrics

**Published:** 1899-07

**Authors:** Maud J. Frye

**Affiliations:** Clinical instructor in diseases of children, Medical Department, University of Buffalo


### Progress in Medical Science.

### PEDIATRICS.

Reported by MAUD J. FRYE, M. D.,
Clinical instructor in diseases of children, Medical Department, University of Buffalo.

### NEPHRITIS COMPLICATING GASTRO-ENTERITIS OF INFANTS.

KOPLIK ( *Med. Record*, April 1, 1899,) discusses the symptomatology, prognosis and treatment of nephritis, complicating acute or subacute gastro-enteritis. He finds nephritis in these cases much more common than ordinarily supposed. In a gastro-enteritis, in which the kidney is only slightly involved, nothing may be found but a slight albuminuria. In the severer forms the patient is restless, the intervals of restlessness being relieved by periods of a sort of stupor, the so-called hydroencephaloid of the older authors. Constant vomiting not relieved by gastric lavage, he regards as often a manifestation of uremia. In marked nephritic infection a form of edema of the skin and subcutaneous tissues is present. In this edema, which disappears as the patient improves, the tissues of the inner side of the thigh, the anterior part of the leg, and the dorsum of the foot are swollen and pit on deep pressure.

The urine in mild cases shows on careful examination a trace of albumin. In severe cases there is more or less complete suppression. A catheter withdraws but a small amount, which deposits urates of ammonium and shows microscopically casts, leucocytes, blood and renal epithelium. The albumin is less in amount than one would find in a similar condition in the adult. The nephritis may appear early or late. Its severity is not dependent on the severity of the primary disorder. The prognosis is good with proper treatment.

*Treatment.*—This is in reality the treatment of all severe forms of gastro-enteritis. Opium in any form, mercury, coal-tar products, designed to disinfect the gastro-intestinal tract, only tend to deepen the toxemia, and by irritating the kidney intensify the functional disturbance in that organ. A full and free supply of water through all possible channels is one of the greatest means of placing these cases on a convalescent basis. Stomach washings and rectal irrigations should be daily employed. For the latter use sterilised water, 1000, sodium chloride 4, sodium carbonate 3; T. 38°--40°C. For rectal irrigation use an adult size stiff rubber tube. Undress the child completely, wrap in a banket and raise the buttocks high. Use a quart or more of the solution and manage to leave considerable in the gut. In very severe cases use 200 c.c. or more of normal salt solution, sterilised, under the skin of the abdomen daily. The only stomach medication to be employed is bismuth. Stop milk, whether a nursling or bottle-fed baby, and give the child albumin water, or beef juice mixed with sterilised barley water. Acorn cocoa is useful. Warm baths with massage in the bath are useful. Strychinin, hypodermatically, may be cautiously employed when need arises.

---

### TREATMENT OF DYSENTERY.

Dr. Christopher C. Cronkhite (*Med. Review*, May 20, 1899,) gives an account of an epidemic of dysentery in which he had an opportunity of treating twenty-three cases. Owing to the bad hygienic conditions prevailing it was found very difficult to successfully combat the disease. The treatment consisted chiefly in the administration of tannigen, in doses of 5 to 10 gr., every three or four hours, according to the age, in connection with the necessary dietetic regulations. In some cases its use was preceded by small doses of calomel, given for the purpose of cleansing the alimentary tract. Under this treatment the fatality in twenty-three cases was only two, and these, the author believes, would have recovered with careful and intelligent nursing. On the ground of two years' observation, he states that in diarrhea, tannigen is his first and last remedy, that it will cure the large majority of cases, and that it can be used with absolute confidence in its powerful curative properties in dysentery and diarrhea.

